# Maternal bisphenol urine concentrations, fetal growth and adverse birth outcomes: A population-based prospective cohort

**DOI:** 10.1186/s12940-021-00747-6

**Published:** 2021-05-15

**Authors:** Chalana M. Sol, Charissa van Zwol - Janssens, Elise M. Philips, Alexandros G. Asimakopoulos, Maria-Pilar Martinez-Moral, Kurunthachalam Kannan, Vincent W. V. Jaddoe, Leonardo Trasande, Susana Santos

**Affiliations:** 1 The Generation R Study Group, Erasmus MC, University Medical Center Rotterdam, PO Box 2040, Rotterdam, 3000 CA the Netherlands; 2 Department of Pediatrics, Erasmus MC – Sophia Children’s Hospital, University Medical Center Rotterdam, Rotterdam, the Netherlands; 3 Wadsworth Center, New York State Department of Health, and Department of Environmental Health Sciences, School of Public Health, State University of New York at Albany, Albany, NY12201 USA; 4 Department of Chemistry, the Norwegian University of Science and Technology (NTNU), NO-7491 Trondheim, Norway; 5 Biochemistry Department, Faculty of Science, King Abdulaziz University, Jeddah, Saudi Arabia; 6 Department of Pediatrics, New York University School of Medicine, New York City, NY 10016 USA; 7 Department of Environmental Medicine, New York University School of Medicine, New York City, NY 10016 USA; 8 Department of Population Health, New York University School of Medicine, New York City, NY USA; 9 New York Wagner School of Public Service, New York City, NY 10016 USA; 10 New York University Global Institute of Public Health, New York City, NY 10016 USA

**Keywords:** Bisphenol, Endocrine disruptor, Fetal growth, Prematurity

## Abstract

**Background:**

Exposure to bisphenols may affect fetal growth and development. The trimester-specific effects of bisphenols on repeated measures of fetal growth remain unknown. Our objective was to assess the associations of maternal bisphenol urine concentrations with fetal growth measures and birth outcomes and identify potential critical exposure periods.

**Methods:**

In a population-based prospective cohort study among 1379 pregnant women, we measured maternal bisphenol A, S and F urine concentrations in the first, second and third trimester. Fetal head circumference, length and weight were measured in the second and third trimester by ultrasound and at birth.

**Results:**

An interquartile range increase in maternal pregnancy-averaged bisphenol S concentrations was associated with larger fetal head circumference (difference 0.18 (95% confidence interval (CI) 0.01 to 0.34) standard deviation scores (SDS), *p*-value< 0.05) across pregnancy. When focusing on specific critical exposure periods, any detection of first trimester bisphenol S was associated with larger second and third trimester fetal head circumference (difference 0.15 (95% CI 0.05 to 0.26) and 0.12 (95% CI 0.02 to 0.23) SDS, respectively) and fetal weight (difference 0.12 (95% CI 0.02 to 0.22) and 0.16 (95% CI 0.06 to 0.26) SDS, respectively). The other bisphenols were not consistently associated with fetal growth outcomes. Any detection of bisphenol S and bisphenol F in first trimester was also associated with a lower risk of being born small size for gestational age (Odds Ratio 0.56 (95% CI 0.38 to 0.74) and 0.55 (95% CI 0.36 to 0.85), respectively). Bisphenols were not associated with risk of preterm birth.

**Conclusions:**

Higher maternal bisphenol S urine concentrations, especially in the first trimester, seem to be related with larger fetal head circumference, higher weight and a lower risk of being small size for gestational age at birth.

**Supplementary Information:**

The online version contains supplementary material available at 10.1186/s12940-021-00747-6.

## Background

Bisphenols are endocrine disrupting chemicals and are widespread in food packaging, thermal paper receipts and personal care products [1–3]. It has been suggested that exposure to bisphenols in pregnancy may adversely affect placental development and function and subsequently lead to suboptimal fetal growth and adverse birth outcomes [4, 5]. However, two meta-analyses of published studies reported no associations of fetal exposure to bisphenol A (BPA) with weight, length or head circumference at birth, while another reported positive associations with birth weight [6–8]. Two of these reported no association between fetal exposure to BPA and gestational age at birth [7, 8]. A few studies assessing the associations of bisphenol exposure with fetal ultrasound measurements showed inconsistent results [9–12]. To fully capture fetal growth variation, analyses on repeated measures of fetal growth are needed. To our knowledge, this has only been assessed in two studies investigating BPA but not its substitutes. A case-control study among 130 preterm children and 352 random term singleton controls found no association of maternal BPA urine concentrations with repeated measures of fetal growth [13]. A prospective study among 470 mother-child pairs showed that maternal BPA urine concentrations was associated with lower fetal femur length and estimated fetal weight growth from 12 until 20 weeks [14]. Due to the short biological half-lives of bisphenols, multiple pools of urines are needed to limit measurement error [15]. The availability of multiple measurements of bisphenol urine concentrations during pregnancy might also allow the study of trimester-specific effects of bisphenols on repeated measures of fetal growth, which would enable identification of critical exposure periods and detection of fetal growth variation that might not be captured with single measurements.

We examined, among 1379 women participating in a population-based cohort study, the associations of bisphenol urine concentrations at three time points in pregnancy, with fetal head circumference, length and weight measured at two time points during pregnancy and at birth and with the risks of preterm birth and being born small size for gestational age.

## Methods

### Study design

This study was embedded in the Generation R Study, a population-based prospective cohort study from fetal life onwards in Rotterdam, the Netherlands [16]. The study was approved by the Medical Ethics Committee of Erasmus MC, University Medical Center in Rotterdam. Written informed consent was obtained from all participants. Bisphenol urine concentrations were measured among a subgroup of 1405 mothers with three urine samples available in pregnancy, whose singleton children also participated in postnatal studies. Women in this subgroup were similar to the broader Generation R cohort in terms of socio-demographic and lifestyle characteristics [17]. We excluded mothers without information on bisphenol urine concentrations in at least one trimester. The population for analysis comprises 1379 pregnant women (specific sample per outcome is given in Supplemental Fig. S1).

### Maternal bisphenol urine concentrations

Bisphenol concentrations were measured in a spot urine sample obtained from each participant at early pregnancy (median 12.9 weeks of gestation, 25th–75th percentiles 12.1–14.5), mid-pregnancy (median 20.4 weeks of gestation, 25th–75th percentiles 20.4–20.9) and late pregnancy (median 30.2 weeks of gestation, 25th–75th percentiles 29.9–30.8). These periods were considered first, second and third trimester. Urine samples were collected between February 2004 and July 2005. Details on collection, transportation, and analysis methodology have been described previously [17]. Briefly, women were asked to urinate into a polypropylene urine collection container during their visit at our research center between 8 a.m. and 8 p.m. This container with the single void urine was then stored at 4 degrees Celcius until at most 24 h later when it was distributed manually into 25-ml polypropylene vials to be frozen at − 20 degrees Celcius. The urine specimens were shipped on dry ice in 4 ml polypropylene vials to the Wadsworth Center, New York State Department of Health, Albany, New York. There, bisphenols were quantitatively detected using a liquid-liquid extraction method, followed by enzymatic deconjugation of the glucuronidated bisphenols coupled with high performance liquid chromatography electrospray ionization-tandem mass spectrometry. Because the molecules were enzymatically digested to release conjugated forms, the concentrations presented here represent the total concentrations of bisphenol A, bisphenol S and bisphenol F. Creatinine concentrations were assessed using high performance liquid chromatography electrospray ionization-tandem mass spectrometry with a limit of detection (LOD) of 0.30 ng/ml. Individual bisphenols were included in the total group and assessed individually if less than 80% of the sample concentrations were below the LOD. Bisphenol concentrations below LOD were substituted by LOD/√2 [18]. We calculated the weighted molar sums for the group representing total bisphenols. To account for urinary dilution, urine concentrations were converted to μg/g creatinine (for separate metabolites) or μmol/g creatinine (for the metabolite group). Descriptive statistics of the bisphenols investigated in each trimester are shown in Table 1. We calculated pregnancy-averaged concentrations based on the first, second and third trimester concentrations. For bisphenol S (BPS) and F (BPF), due to > 80% of concentrations below LOD in one trimester, pregnancy-averaged concentrations were calculated based on urine concentrations in two trimesters.

**Table 1:** Urine bisphenol concentrations during pregnancy (*n* = 1379)

	LOD	First trimester		Second trimester		Third trimester	
		Median (25th, 75th percentile)	% below LOD	Median (25th, 75th percentile)	% below LOD	Median (25th, 75th percentile)	% below LOD
**Total bisphenols**		9.22 (3.53, 20.29)		6.35 (3.07, 14.01)		10.11 (4.67, 20.00)	
Bisphenol A	0.66	4.93 (1.10, 12.36)	21.0	5.83 (2.71, 13.05)	6.9	6.58 (2.70, 13.24)	10.1
Bisphenol S	0.20	0.68 (0.13, 2.42)	32.1	0.13 (0.13, 0.39)	70.6	NA	> 80%
Bisphenol F	0.90	0.62 (0.62, 2.04)	59.9	NA	> 80%	0.62 (0.62, 2.58)	70.9

Absolute urine concentrations of individual and grouped bisphenols and the limits of detection are expressed in nmol/L urine

*LOD* limit of detection, *NA* not applicable due to > 80% of concentrations below the limit of detection

### Fetal growth measures and birth outcomes

In second and third trimester, we measured fetal head circumference and femur length to the nearest millimeter using standardized ultrasound procedures [19]. Estimated fetal weight was calculated using the formula of Hadlock et al. [20] Gestational age at time of growth measurements in second and third trimester was based on the first trimester ultrasound [21]. Gestational-age-adjusted standard deviation scores (SDS) for each measurement were calculated using reference growth curves derived in the same cohort as the current study [21]. Information on gestational age, head circumference, length and weight at birth was obtained from medical records. Since head circumference and length were not routinely measured in each delivery center, fewer measurements were available (Supplemental Fig. S1). We created sex- and gestational age-adjusted SDS for weight, length and head circumference at birth based on North European reference charts [22]. The median (95% range) gestational ages for second and third trimester ultrasounds and birth were 20.4 (18.9, 22.7), 30.4 (28.7, 32.6), and 40.3 (36.7, 42.3) weeks, respectively. Preterm birth was defined as < 37 weeks of gestation. Small size for gestational age at birth was defined in the whole Generation R study population as sex- and gestational age-adjusted birth weight < 10th percentile (− 1.41 SD). Appropriate size for gestational age at birth was used as the reference group.

### Covariates

We obtained information on maternal age, parity, educational level, ethnicity, pre-pregnancy weight, folic acid supplement use, smoking habits and alcohol consumption from questionnaires during pregnancy. At enrollment, maternal height was measured and pre-pregnancy body mass index (BMI, kg/m^2^) was calculated. Maternal daily caloric intake was estimated from a validated food frequency questionnaire filled in at enrollment, covering average intake in first trimester [23, 24].

### Statistical analysis

After substitution of concentrations below the LOD with LOD/√2, we natural log-transformed the creatinine-adjusted bisphenol urine concentrations to reduce variability and account for right skewedness of the distribution and standardized by the interquartile range to ease interpretation of effect sizes. We assessed Pearson correlations between all bisphenols. We performed linear mixed effects models to assess associations of the average bisphenol urine concentrations over pregnancy with head circumference, length or weight SDS repeatedly measured in second and third trimester and at birth. We inspected the pattern of associations using generalized additive models, and no clear non-linearity was observed. All models included a random intercept and slope. We additionally included an interaction term between pregnancy-averaged bisphenol urine concentrations and gestational age to allow the exposure-outcome association to change across pregnancy. To identify potential critical exposure periods, we assessed associations of bisphenol concentrations in first, second and third trimester with outcome measurements in second and third trimester and at birth using linear regression models. For these analyses, we examined only outcomes at the same or subsequent time points as the exposure. Due to the high percentage of concentrations the below LOD for BPS and BPF, we first performed these models using the bisphenols categorized as detected (above the LOD) and undetected (below the LOD) and then repeated these models using continuous bisphenol concentrations as a sensitivity analysis. We performed binary logistic regression models to assess the associations of detection of bisphenols in first, second and third trimester with risks of preterm birth and small size for gestational age at birth. Then, we repeated these models using continuous levels of bisphenols during first, second and third trimester and pregnancy-averaged bisphenol urine concentrations. Due to the low number of preterm births and potential lack of power, as a sensitivity analysis, we performed linear regression models to assess the associations of bisphenol urine concentrations, categorized as detected and undetected and continuously, with gestational age at birth. To examine the independent associations of maternal first, second and third trimester bisphenol concentrations, we simultaneously included in one model the exposures during all three trimesters, creating a mutually adjusted model. Potential confounders were identified based on graphical criteria for confounding by visualizing a directed acyclic graph (Supplemental Fig. S2 presents a simplified version) and were included in the models if they changed the effect estimates > 10% for at least one outcome [25]. Since the mechanism of action of endocrine disrupting chemicals may be sex-specific and influenced by folic acid, we tested for statistical interaction of bisphenol urine concentrations with fetal sex and folic acid supplement use [26, 27]. We did not find statistically significant interactions (*p*-values> 0.10) and no further stratified analyses were performed. To maintain statistical power and reduce bias related to missing data on covariates, we performed multiple imputation according to the Markov Chain Monte Carlo method. The percentage of missing values for covariates ranged from 0 to 25%. Ten imputed datasets were created and no substantial differences were found between original and imputed datasets. To correct for multiple hypothesis testing, each *p*-value was compared with a threshold defined as 0.05 divided by the effective number of independent tests estimated based on the correlation between the exposures (*p*-value threshold of 0.020) [28]. We present results based on pooled imputed datasets. Statistical analyses were performed using Statistical Package of Social Sciences version 25.0 for Windows (SPSS Inc., Chicago, IL, USA) and R software (version 3.6.1). We used the software package *nlme* for the linear mixed effects models.

## Results

Table 2 shows participants characteristics. Of the 1379 pregnant women included, mean maternal age was 30.5 years, 7.4% were lower educated, 61.9% were European and median pre-pregnancy BMI was 22.7 kg/m^2^. In total, 2.5% of all women had a preterm delivery. We observed low correlations between bisphenol urine concentrations across pregnancy and between individual bisphenols within the same trimester (Supplemental Table S1).

**Table 2:** Characteristics of participants (n = 1379)

	Total group (***n*** = 1379)
**Maternal characteristics**	
Age, mean (SD), years	30.5 (4.8)
Parity, No. nulliparous (%)	839 (61.2)
Educational level, No. low (%)	102 (7.4)
Ethnicity, No. European (%)	846 (61.9)
Folic acid supplement use, No. yes (%)	887 (80.6)
Smoking, No. nonsmoking (%)	939 (75.5)
First trimester, No. nonsmoking (%)	952 (78.4)
Second trimester, No. nonsmoking (%)	1055 (88.7)
Third trimester, No. nonsmoking (%)	1050 (89.3)
Alcohol consumption, No. no alcohol use (%)	527 (42.6)
First trimester, No. no alcohol use (%)	616 (50.5)
Second trimester, No. no alcohol use (%)	777 (65.6)
Third trimester, No. no alcohol use (%)	747 (63.5)
Pre-pregnancy body mass index, median (95% range), kg/m^2^	22.7 (18.5, 34.9)
Daily caloric intake, mean (SD), kcal	2078.1 (511.2)
**Fetal growth characteristics**	
*Second trimester*	
Gestational age, median (95% range), weeks	20.4 (18.9, 22.7)
Head circumference, mean (SD), mm	178.0 (12.0)
Femur length, mean (SD), mm	33.2 (2.9)
Estimated fetal weight, mean (SD), g	369.2 (72.7)
*Third trimester*	
Gestational age, median (95% range), weeks	30.4 (28.7, 32.6)
Head circumference, mean (SD), mm	285.9 (11.5)
Femur length, mean (SD), mm	57.5 (2.8)
Estimated fetal weight, mean (SD), g	1620.5 (236.5)
**Birth characteristics**	
Males, No. (%)	696 (50.5)
Gestational age, median (95% range), weeks	40.3 (36.7, 42.3)
Birth weight, mean (SD), g	3453.9 (498.5)
Birth length, mean (SD), cm	50.3 (2.3)
Birth head circumference, mean (SD), cm	33.8 (1.7)
Preterm birth, No. (%)	35 (2.5)
Small size for gestational age, No. (%)	114 (8.3)

Values are observed data and represent means (SD), medians (95% range), or number of subjects (valid %). *SD* standard deviation

Table 3 shows that an interquartile range increase in maternal pregnancy-averaged BPS urine concentrations was associated with larger fetal head circumference (difference 0.18 (95% confidence interval (CI) 0.01 to 0.34) SDS). This association did not differ by timing of outcome measurement (*p*-value for interaction with gestational age = 0.15). No associations were observed for maternal pregnancy-averaged bisphenol concentrations with fetal length and weight. Basic models are shown in Supplemental Table S2. When comparing women with detected and undetected bisphenol concentrations and focusing on potential critical exposure periods, we observed that any detection of first trimester BPS was associated with larger second and third trimester fetal head circumference (difference 0.15 (95% CI 0.05 to 0.26) and 0.12 (95% CI 0.02 to 0.23) SDS, respectively) and fetal weight (difference 0.12 (95% CI 0.02 to 0.22) and 0.16 (95% CI 0.06 to 0.26) SDS, respectively) (Table 4). These associations remained significant after multiple testing correction. Detection of BPF in third trimester was associated with higher length SDS at birth while detection of BPF in first trimester was associated with higher weight SDS during third trimester and at birth. These associations did not remain significant after correction for multiple testing. When continuous bisphenol concentrations were assessed, associations in the same direction but with slightly attenuated effect estimates were observed for BPS and BPF (Supplemental Table S3). No associations were found for total bisphenols and BPA with the measures of fetal growth (Supplemental Table S3 and S4). Basic models are shown in Supplemental Table S5.

**Table 3:** Maternal pregnancy-averaged bisphenol concentrations and fetal growth measures

	Fetal head circumference		Fetal length		Fetal weight	
	Effect estimate, SDS (95% Confidence Interval)	***p***-value for interaction with gestational age	Effect estimate, SDS (95% Confidence Interval)	***p***-value for interaction with gestational age	Effect estimate, SDS (95% Confidence Interval)	***p***-value for interaction with gestational age
**Total bisphenols**	− 0.10 (− 0.25, 0.06)	0.35	0.04 (− 0.10, 0.18)	0.59	0.06 (− 0.06, 0.18)	0.35
**Bisphenol A**	−0.12 (− 0.27, 0.03)	0.22	0.03 (− 0.10, 0.17)	0.77	0.06 (− 0.06, 0.17)	0.25
**Bisphenol S**	0.18 (0.01, 0.34)*	0.15	0.01 (−0.15, 0.16)	0.93	0.05 (− 0.08, 0.18)	0.89
**Bisphenol F**	−0.05 (− 0.22, 0.12)	0.65	− 0.13 (− 0.29, 0.02)	0.13	− 0.04 (− 0.17, 0.09)	0.40

Main effects and *p*-values for interaction with gestational age at growth measurement obtained from linear mixed effects models. Main effects reflect the difference in fetal growth measures in SDS for an interquartile range increase in each natural log-transformed pregnancy-averaged bisphenol (in μmol/g creatinine). Models include fetal sex, maternal age, pre-pregnancy body mass index, educational level, ethnicity, parity, smoking habits, alcohol consumption, daily caloric intake, folic acid supplement use, and gestational age at growth measurement. Models include an interaction term between exposure concentration and gestational age at growth measurement, a random intercept for each participant, and a random slope for gestational age at growth measurement. *p-value< 0.05

*SDS* standard deviation scores

**Table 4:** Maternal trimester-specific bisphenol concentrations (categorized as detected and undetected) and fetal growth measures

	Fetal head circumference Difference in SDS (95% Confidence Interval)			Fetal length Difference in SDS (95% Confidence Interval)			Fetal weight Difference in SDS (95% Confidence Interval)		
	Second trimester	Third trimester	Birth	Second trimester	Third trimester	Birth	Second trimester	Third trimester	Birth
**Bisphenol A**									
First trimester	− 0.04 (− 0.16, 0.08)	− 0.06 (− 0.18, 0.06)	0.02 (− 0.19, 0.22)	0.11 (− 0.01, 0.23)	0.05 (− 0.07, 0.17)	0.02 (− 0.16, 0.20)	0.04 (− 0.07, 0.15)	−0.02 (− 0.14, 0.10)	0.02 (− 0.10, 0.15)
Second trimester	−0.01 (− 0.21, 0.18)	−0.03 (− 0.22, 0.16)	−0.04 (− 0.36, 0.28)	0.03 (− 0.16, 0.22)	−0.01 (− 0.20, 0.18)	−0.13 (− 0.41, 0.15)	−0.05 (− 0.23, 0.13)	−0.04 (− 0.23, 0.15)	0.05 (− 0.15, 0.24)
Third trimester		− 0.08 (− 0.24, 0.08)	−0.04 (− 0.31, 0.22)		0.01 (− 0.15, 0.17)	0.19 (− 0.05, 0.43)		0.03 (− 0.13, 0.19)	0.01 (− 0.16, 0.17)
**Bisphenol S**									
First trimester	0.15 (0.05, 0.26)†	0.12 (0.02, 0.23)†	0.11 (−0.06, 0.29)	0.10 (−0.01, 0.20)	0.09 (− 0.02, 0.19)	0.08 (− 0.07, 0.23)	0.12 (0.02, 0.22)†	0.16 (0.06, 0.26)†	0.10 (− 0.00, 0.21)
Second trimester	0.09 (−0.02, 0.20)	0.06 (− 0.05, 0.16)	0.03 (− 0.15, 0.21)	0.02 (− 0.08, 0.12)	0.02 (− 0.09, 0.13)	0.11 (− 0.05, 0.26)	−0.04 (− 0.14, 0.06)	0.01 (− 0.10, 0.11)	0.08 (− 0.02, 0.19)
Third trimester		NA	NA		NA	NA		NA	NA
**Bisphenol F**									
First trimester	0.02 (−0.08, 0.12)	0.06 (−0.04, 0.16)	− 0.06 (− 0.23, 0.11)	0.03 (− 0.07, 0.12)	0.08 (− 0.02, 0.18)	0.04 (− 0.11, 0.18)	0.07 (− 0.03, 0.16)	0.10 (0.00, 0.20)*	0.10 (0.00, 0.20)*
Second trimester	NA	NA	NA	NA	NA	NA	NA	NA	NA
Third trimester		0.02 (−0.09, 0.13)	−0.05 (− 0.32, 0.13)		0.03 (− 0.08, 0.14)	0.16 (0.00, 0.31)*		0.07 (− 0.04, 0.17)	0.08 (− 0.02, 0.19)

Values are regression coefficients (95% confidence intervals) that reflect the differences in fetal head circumference, length and weight in SDS in the second and third trimester and at birth for detected bisphenol concentrations, as compared to the reference group (undetected concentrations). Models include fetal sex (except in the models for the outcomes at birth), maternal age, pre-pregnancy body mass index, educational level, ethnicity, parity, smoking habits and alcohol consumption during each trimester, daily caloric intake and folic acid supplement use. *p-value< 0.05; †*p*-value< 0.020

*NA* not applicable due to > 80% of concentrations below the limit of detection, *SDS* standard deviation scores

Trimester-specific and pregnancy-averaged total bisphenols and BPA, BPS and BPF, either categorized as detected and undetected or continuously, were not associated with risk of preterm birth (Table 5 and Supplemental Table S6). Any detection of BPS and BPF in first trimester was associated with a lower risk of being born small size for gestational age (Odds Ratio 0.56 (95% CI 0.38 to 0.84) and 0.55 (95% CI 0.36 to 0.85), respectively) (Table 5). These associations remained significant after multiple testing correction. When assessing the bisphenols continuously, an interquartile range increase in maternal first trimester BPS concentrations was associated with a lower risk of small size for gestational age at birth, although this association was no longer significant after multiple testing correction (Supplemental Table S6). No associations were observed for the other bisphenols with the risk of small size for gestational age at birth. Also, no associations were observed for total bisphenols and BPA with the risk of preterm birth or being born small size for gestational age in the mutually adjusted models (Supplemental Table S7). Results from the unadjusted models are shown in Supplemental Table S8. There were no associations of bisphenols, either categorized as detected and undetected or continuously, with gestational age at birth continuously (Supplemental Table S9).

**Table 5:** Maternal trimester-specific bisphenol concentrations (categorized as detected and undetected) and preterm birth and small size for gestational age

	Preterm birth Odds Ratio (95% Confidence Interval)			Small size for gestational age at birth Odds Ratio (95% Confidence Interval)		
	First trimester	Second Trimester	Third trimester	First trimester	Second Trimester	Third trimester
**Bisphenol A**	1.62 (0.76, 3.17)	1.90 (0.65, 5.58)	1.20 (0.41, 3.47)	0.91 (0.56, 1.46)	1.02 (0.47, 2.20)	1.08 (0.56, 2.09)
**Bisphenol S**	1.63 (0.84, 3.24)	0.89 (0.43, 1.84)	NA	0.56 (0.38, 0.84)†	0.91 (0.59, 1.42)	NA
**Bisphenol F**	1.14 (0.56, 2.29)	NA	1.01 (0.48, 2.12)	0.55 (0.36, 0.85)†	NA	0.87 (0.56, 1.36)

Values are odds ratios (95% confidence intervals) from binary logistic regression models that reflect the risk of preterm birth and small size for gestational age at birth for detected bisphenol concentrations, as compared to the reference group (undetected concentrations). Models include maternal age, pre-pregnancy body mass index, ethnicity, educational level, parity, folic acid supplement use, alcohol consumption and smoking habits during each trimester or during pregnancy and daily caloric intake. †*p*-value< 0.020

*NA* not applicable due to > 80% of concentrations below the limit of detection

## Discussion

We observed, in a population-based prospective cohort study, that higher maternal BPS urine concentrations, especially in first trimester, were associated with larger fetal head circumference and higher weight and lower risk of small size for gestational age at birth. No consistent associations were observed for the other bisphenols. Bisphenols are used in many consumer products [1–3]. Fetal exposure to bisphenols may adversely affect placental formation and subsequently early growth and development, which might have persistent health effects [5]. Due to concerns about these possible adverse effects of BPA, it is increasingly being replaced by other bisphenols such as BPS and BPF. However, it is unclear whether these replacements have fewer adverse health effects than BPA.

Of the three meta-analyses concerning BPA and birth outcomes, two reported no consistent associations of fetal exposure to BPA with weight, length or head circumference at birth, while one reported positive associations with birth weight [6–8]. Two studies evaluated maternal BPA urine concentrations during pregnancy with repeated measures of fetal growth. A case-control study from the USA among 130 preterm children and 352 random term singleton controls found no association of cumulative BPA exposure during pregnancy with repeated measures of fetal growth [13]. However, a prospective study among 470 mother-child pairs from Spain showed an association of average maternal BPA urine concentrations with lower fetal femur length and estimated fetal weight growth from 12 until 20 weeks [14]. We observed that higher maternal third trimester BPA urine concentrations were associated with smaller head circumference in the third trimester but no associations were observed for length and weight. These results have to be interpreted with caution, since the association of BPA with fetal head circumference was no longer significant after multiple testing correction. Two previous meta-analyses reported no association between fetal exposure to BPA and gestational age at birth [7, 8]. Similarly, in our study, maternal pregnancy-averaged and trimester-specific bisphenol concentrations were not associated with the risk of preterm birth or with gestational age at birth.

Previous studies assessing the associations of maternal BPS urine concentrations with fetal growth measures during pregnancy and at birth showed inconsistent results [9, 11, 12, 29–31]. In two prospective cohort studies among Chinese pregnant women, no associations were found between maternal BPS urine concentrations and ultrasound parameters of fetal growth during third trimester and birth weight or length [9, 32]. In a nested case-control study among 476 US pregnant women, detection of BPS in maternal urine was associated with lower femur length and estimated fetal weight across pregnancy and with lower birth weight among males [12]. In another prospective cohort study among 845 Chinese pregnant women, higher BPS urine concentrations were associated with lower birth weight, birth length and ponderal index [29]. On the other hand, in a prospective cohort study among 867 pregnant women from Puerto Rico, higher average urine concentrations of BPS during mid-pregnancy were associated with an increased risk of being large for gestational age at birth [31]. In the current study, higher maternal BPS concentrations, especially in the first trimester, were associated with larger fetal head circumference and higher weight and a lower risk of small size for gestational age at birth, suggesting that BPS exposure enhances fetal growth. We did not hypothesize this association beforehand, although it has been suggested previously that exposure to BPS during pregnancy influences maternal hormone levels, which could influence fetal growth differently based on the timing of exposure [33, 34]. In our study, the detection frequency for BPS was much lower in the second trimester than in the first trimester, which might have masked any effects of exposure during second trimester. Also, due to the high percentage of concentrations below the LOD, we were not able to study the associations for third trimester BPS.

Two studies investigating maternal BPF urine concentrations during pregnancy showed an inverse relationship with birth weight [29, 30]. Three studies showed no association of BPF with birth weight, as in our study [9, 11, 31]. One study showed decreased odds of preterm birth with higher BPF urine concentrations during pregnancy [31]. In our study, an association of first trimester maternal urine BPF concentrations was only found in the sensitivity analysis.

The availability of repeatedly assessed maternal bisphenol urine concentrations during pregnancy enabled us to investigate windows of vulnerability to exposure. We observed differences in associations according to trimester of exposure, suggesting that first trimester might be a particularly sensitive window to exposure. The observed associations might be mediated by the rapid placental development that occurs during early to mid-pregnancy. BPA inhibits proliferation and invasion of human trophoblast cells [35–37], and BPS can interfere with trophoblast fusion in the placenta and cause maternal endocrine dysfunction [34, 38]. Whether these mechanisms explain our observed associations needs to be further studied. The effect estimates observed in the current study are small and thus may lack clinical relevance. However, even small differences in fetal growth may be crucial for health outcomes in later life. Since most associations were no longer significant after multiple testing correction, we cannot exclude the possibility of these results being chance findings. Also, bisphenol concentrations in our study are comparable to the concentrations reported in previously published studies and thus differences in results across studies are not explained by differences in exposure levels [9, 13].

The major strengths of this study were the large sample size, the population-based cohort design from early life onwards, the availability of three urine measurements of bisphenol concentrations, and the use of repeated fetal growth measures during pregnancy and at birth. No substantial differences were observed between women in this subgroup and in the broader cohort and thus, although it cannot be excluded, selection bias seems unlikely [17]. In this study, we observed high within-individual variability for bisphenols across pregnancy, which might reflect their short biological half-lives [39]. Thus, measurement error may have led to an underestimation of the trimester-specific effect estimates. Single void urine samples collected during the appointment at our research facility were used to assess exposure. The timing of urine collection might have influenced our exposure assessment, due to the short biological half-lives of bisphenols and the fact that exposure is mainly influenced by behavior, which differs throughout the day. This might have led to non-differential error and thus underestimation of the effect sizes. The effect estimates for the pregnancy-averaged associations may be less affected by measurement error, but may lack interpretability if trimester-specific effects are present. For BPS, the pregnancy-averaged concentrations mainly represent the first trimester, due to the high percentage of concentrations below the LOD in the second and third trimester. Taking this into account, we would expect similar associations for pregnancy-averaged and first trimester BPS with fetal growth measures and birth outcomes. We observed associations in the same direction, although the magnitude of the associations for the pregnancy-averaged concentrations was attenuated. The high percentage of concentrations below LOD precluded the study of the associations for BPS in third trimester and BPF in second trimester. Additionally, to avoid the influence of the in the associations for BPS in second trimester and BPF in first and third trimester, we have performed the analyses categorizing bisphenols as detected and undetected. This showed comparable results to the continuous analyses. Although we adjusted for a large number of confounders, residual confounding due to unmeasured lifestyle variables might still be present. Diet is a major source of bisphenol exposure, and thus represents a potential confounder in these associations. We adjusted for total caloric intake in our models, but residual confounding by diet might still be present. From the current observational data, no conclusions can be drawn on the causality and mechanisms underlying the observed associations.

## Conclusions

Fetal exposure to BPS, especially in the first trimester, seems to be related with larger fetal head circumference and higher weight and a lower risk of small size for gestational age at birth. BPA, BPS and BPF were not associated with the risk of preterm birth. Future studies in contemporary cohorts with possibly higher exposure levels are needed to further investigate the associations of newer bisphenols with fetal growth and adverse birth outcomes and to clarify the importance of the first trimester as a particularly sensitive window to exposure.

## Supplementary Information

**Additional file 1: Supplemental Material Bisphenols**. Tables containing additional subscriptive data and results of sensitivity analysis.

## Data Availability

The datasets generated and/or analysed during the current study are not publicly available, but are available from the corresponding author on reasonable request.

## Abbreviations

BMI: Body mass index
BPA: Bisphenol A
BPF: Bisphenol F
BPS: Bisphenol S
CI: Confidence interval
LOD: Limit of detection
NA: Not applicable
SD: Standard deviation
SDS: Standard deviation scores
