# Supplementary material for: Maternal bisphenol urine concentrations, fetal growth and adverse birth outcomes: A population-based prospective cohort
Source: Environ Health. 2021 May 15;20:60. doi: 10.1186/s12940-021-00747-6 (PMC8126069; doi:10.1186/s12940-021-00747-6)
Supplement: Supplementary file 1 — Additional file 1: Supplemental Material Bisphenols. Tables containing additional subscriptive data and results of sensitivity analysis. [file 12940_2021_747_MOESM1_ESM.docx]

**Supplemental Material**

**Maternal bisphenol urine concentrations, fetal growth and adverse birth outcomes. A population-based prospective cohort.**

Chalana M Sol, MD, Charissa van Zwol - Janssens, MD, Elise M Philips, MD, Alexandros G Asimakopoulos, PhD, Maria-Pilar Martinez-Moral, PhD, Kurunthachalam Kannan, PhD, Vincent WV Jaddoe, MD, PhD, Leonardo Trasande, MD, MPP*, Susana Santos, PhD*

**Authors contributed equally*

**Corresponding author:** Vincent WV Jaddoe MD PhD, The Generation R Study Group (Na 29-08), Erasmus MC, University Medical Center Rotterdam, PO Box 2040, 3000 CA, Rotterdam, The Netherlands. Phone: +31 10 7043405, fax: +31 10 70 44645, e-mail address: [v.jaddoe@erasmusmc.nl](mailto:v.jaddoe@erasmusmc.nl).

**CONTENTS**

**Supplemental Table S1.** Pearson’s correlations between natural log transformed non-creatinine adjusted maternal urine bisphenol concentrations

**Supplemental Table S2.** Maternal pregnancy-averaged bisphenol concentrations and fetal growth measures

**Supplemental Table S3.** Maternal trimester-specific bisphenol concentrations and fetal growth measures

**Supplemental Table S4.** Maternal trimester-specific bisphenols concentrations and fetal growth measures, mutually adjusted model

**Supplemental Table S5.** Maternal trimester-specific bisphenol concentrations (categorized as detected and undetected) and fetal growth measures

**Supplemental Table S6.** Maternal pregnancy-averaged and trimester-specific bisphenols concentrations and preterm birth and small size for gestational age

**Supplemental Table S7.** Maternal trimester-specific bisphenol concentrations and preterm birth and small size for gestational age at birth, mutually adjusted model

**Supplemental Table S8.** Maternal trimester-specific bisphenol concentrations (categorized as detected and undetected) and preterm birth and small size for gestational age at birth

**Supplemental Table S9.** Maternal pregnancy-averaged and trimester-specific bisphenol concentrations and gestational age at birth

**Supplemental Figure S1.** Selection of study participants

**Supplemental Figure S2.** Directed acyclic graph depicting the relationship between exposure to bisphenols, fetal growth and birth outcomes and covariates

**Supplemental Table S1.** Pearson’s correlations between natural log transformed non-creatinine adjusted maternal urine bisphenol concentrations

|  | **First**  **trimester total bisphenols** | **Second trimester total bisphenols** | **Third trimester total bisphenols** | **First trimester BPA** | **Second trimester BPA** | **Third trimester BPA** | **First trimester BPS** | **Second trimester BPS** | **First trimester BPF** | **Third trimester BPF** |
| --- | --- | --- | --- | --- | --- | --- | --- | --- | --- | --- |
| **First trimester total bisphenols** | 1 | 0.07 | 0.04 | 0.87 | 0.07 | 0.07 | 0.46 | 0.02 | 0.49 | -0.03 |
| **Second trimester total bisphenols** |  | 1 | 0.06 | 0.09 | 0.99 | 0.08 | -0.02 | 0.24 | 0.06 | -0.01 |
| **Third trimester total bisphenols** |  |  | 1 | 0.05 | 0.06 | 0.87 | 0.02 | 0.01 | -0.01 | 0.41 |
| **First trimester BPA** |  |  |  | 1 | 0.09 | 0.07 | 0.17 | 0.03 | 0.28 | -0.02 |
| **Second trimester BPA** |  |  |  |  | 1 | 0.08 | -0.02 | 0.13 | 0.06 | -0.01 |
| **Third trimester BPA** |  |  |  |  |  | 1 | 0.01 | 0.01 | 0.02 | 0.04 |
| **First trimester BPS** |  |  |  |  |  |  | 1 | 0.02 | 0.11 | 0.03 |
| **Second trimester BPS** |  |  |  |  |  |  |  | 1 | -0.01 | 0.03 |
| **First trimester BPF** |  |  |  |  |  |  |  |  | 1 | -0.06 |
| **Third trimester BPF** |  |  |  |  |  |  |  |  |  | 1 |

BPA, bisphenol A; BPF, bisphenol F; BPS, bisphenol S.

**Supplemental Table S2.** Maternal pregnancy-averaged bisphenol concentrations and fetal growth measures

Main effects and p-values for interaction with gestational age at growth measurement obtained from linear mixed effects models. Main effects reflect the difference in fetal growth measures in SDS for an interquartile range increase in each natural log-transformed pregnancy-averaged bisphenols (in µmol/g creatinine). Models include fetal sex and gestational age at growth measurement. Models include an interaction term between exposure concentration and gestational age at growth measurement, a random intercept for each participant, and a random slope for gestational age at growth measurement. *p-value<0.05

SDS, standard deviation scores.

|  | **Fetal head circumference** | |  | **Fetal length** | |  | **Fetal weight** | |  | |
| --- | --- | --- | --- | --- | --- | --- | --- | --- | --- | --- |
|  | **Effect estimate, SDS (95% Confidence Interval)** | **p-value for interaction with gestational age** | | **Effect estimate, SDS (95% Confidence Interval)** | **p-value for interaction with gestational age** | | **Effect estimate, SDS (95% Confidence Interval)** | **p-value for interaction with gestational age** | |  |
| **Total bisphenols** | -0.10 (-0.25, 0.05) | 0.32 | | 0.04 (-0.10, 0.18) | 0.60 | | 0.06 (-0.05, 0.18) | 0.35 | |  |
| **Bisphenol A** | -0.13 (-0.28, 0.02) | 0.20 | | 0.03 (-0.10, 0.17) | 0.78 | | 0.06 (-0.05, 0.17) | 0.25 | |  |
| **Bisphenol S** | 0.18 (0.02, 0.34)* | 0.15 | | 0.01 (-0.14, 0.16) | 0.92 | | 0.06 (-0.06, 0.19) | 0.89 | |  |
| **Bisphenol F** | -0.03 (-0.20, 0.14) | 0.65 | | -0.13 (-0.29, 0.03) | 0.12 | | -0.03 (-0.16, 0.10) | 0.40 | |  |

**Supplemental Table S3.** Maternal trimester-specific bisphenol concentrations and fetal growth measures

|  | **Fetal head circumference**  **Difference in SDS (95% Confidence Interval)** | | |  | **Fetal length**  **Difference in SDS (95% Confidence Interval)** | | |  | | **Fetal weight**  **Difference in SDS (95% Confidence Interval)** | | | |  |
| --- | --- | --- | --- | --- | --- | --- | --- | --- | --- | --- | --- | --- | --- | --- |
|  | **Second trimester** | **Third trimester** | **Birth** | **Second trimester** | | **Third trimester** | **Birth** | | **Second trimester** | | **Third trimester** | **Birth** |  |  |
| **Total bisphenols** |  |  |  |  | | | | |  | | | |  |  |
| First trimester | 0.03 (-0.04, 0.09) | -0.02 (-0.08, 0.05) | 0.06 (-0.05, 0.17) | 0.04 (-0.02, 0.10) | | 0.04 (-0.03, 0.10) | 0.06 (-0.03, 0.16) | | 0.04 (-0.02, 0.10) | | 0.03 (-0.03, 0.09) | 0.02 (-0.05, 0.08) |  |  |
| Second trimester | -0.02 (-0.08, 0.04) | -0.03 (-0.09, 0.03) | -0.01 (-0.11, 0.09) | 0.02 (-0.04, 0.08) | | -0.03 (-0.09, 0.03) | -0.04 (-0.13, 0.05) | | -0.01 (-0.07, 0.04) | | -0.01 (-0.07, 0.05) | -0.03 (-0.09, 0.03) |  |  |
| Third trimester |  | -0.05 (-0.12, 0.01) | -0.04 (-0.15, 0.06) |  | | -0.03 (-0.09, 0.03) | -0.01 (-0.10, 0.08) | |  | | 0.00 (-0.06, 0.06) | -0.04 (-0.10, 0.03) |  |  |
| **Bisphenol A** |  |  |  |  | |  |  | |  | |  |  |  |  |
| First trimester | 0.00 (-0.06, 0.07) | -0.03 (-0.10, 0.03) | 0.07 (-0.03, 0.18) | 0.06 (-0.01, 0.12) | | 0.06 (-0.01, 0.12) | 0.08 (-0.01, 0.18) | | 0.03 (-0.03, 0.09) | | 0.02 (-0.04, 0.08) | 0.00 (-0.06, 0.07) |  |  |
| Second trimester | -0.03 (-0.09, 0.03) | -0.03 (-0.09, 0.03) | 0.00 (-0.10, 0.10) | 0.02 (-0.04, 0.07) | | 0.02 (-0.04, 0.07) | -0.04 (-0.12, 0.05) | | -0.01 (-0.07, 0.04) | | -0.01 (-0.07, 0.04) | -0.03 (-0.09, 0.03) |  |  |
| Third trimester |  | -0.06 (-0.12,  -0.00)* | -0.03 (-0.13, 0.08) |  | | -0.04 (-0.10, 0.02) | 0.03 (-0.06, 0.11) | |  | | -0.01 (-0.07, 0.05) | -0.05 (-0.11, 0.01) |  |  |
| **Bisphenol S** |  |  |  |  | |  |  | |  | |  |  |  |  |
| First trimester | 0.10 (0.03, 0.17)† | 0.05 (-0.03, 0.11) | 0.04 (-0.08, 0.16) | 0.04 (-0.03, 0.11) | | 0.02 (-0.05, 0.09) | 0.01 (-0.09, 0.11) | | 0.07 (0.01, 0.14)* | | 0.07 (0.00, 0.14)* | 0.05 (-0.02, 0.12) |  |  |
| Second trimester | 0.06 (0.00, 0.12)* | 0.04 (-0.02, 0.10) | 0.03 (-0.07, 0.12) | -0.01 (-0.06, 0.05) | | -0.01 (-0.07, 0.05) | 0.01 (-0.08, 0.09) | | -0.03 (-0.09, 0.02) | | 0.00 (-0.05, 0.06) | 0.01 (-0.05, 0.07) |  |  |
| Third trimester |  | NA | NA |  | | NA | NA | |  | | NA | NA |  |  |
| **Bisphenol F** |  |  |  |  | |  |  | |  | |  |  |  |  |
| First trimester | 0.00 (-0.06, 0.07) | 0.02 (-0.04, 0.09) | -0.01 (-0.13, 0.10) | -0.01 (-0.08, 0.06) | | 0.02 (-0.05, 0.09) | 0.00 (-0.10, 0.10) | | 0.00 (-0.06, 0.06) | | 0.06 (-0.01, 0.12) | 0.01 (-0.06, 0.08) |  |  |
| Second trimester | NA | NA | NA | NA | | NA | NA | | NA | | NA | NA |  |  |
| Third trimester |  | 0.00 (-0.07, 0.07) | 0.01 (-0.10, 0.13) |  | | -0.01 (-0.08, 0.06) | 0.10 (-0.09, 0.11) | |  | | 0.02 (-0.04, 0.09) | 0.02 (-0.04, 0.09) |  |  |

Values are regression coefficients (95% confidence intervals) that reflect the differences in fetal head circumference, length and weight in SDS in the second and third trimester and at birth for an interquartile range increase in each natural log-transformed trimester-specific bisphenols (in µmol/g creatinine). Models include fetal sex (except in the models for the outcomes at birth), maternal age, pre-pregnancy body mass index, educational level, ethnicity, parity, smoking habits and alcohol consumption during each trimester, daily caloric intake and folic acid supplement use. *p-value<0.05; †p-value<0.020

NA, not applicable due to >80% of concentrations below the limit of detection; SDS, standard deviation scores.

**Supplemental Table S4.** Maternal trimester-specific bisphenol concentrations and fetal growth measures, mutually adjusted model

|  | **Fetal head circumference**  **Difference in SDS (95% Confidence Interval)** | | |  | **Fetal length**  **Difference in SDS (95% Confidence Interval)** | | |  | | **Fetal weight**  **Difference in SDS (95% Confidence Interval)** | | | |  |
| --- | --- | --- | --- | --- | --- | --- | --- | --- | --- | --- | --- | --- | --- | --- |
|  | **Second trimester** | **Third trimester** | **Birth** | **Second trimester** | | **Third trimester** | **Birth** | | **Second trimester** | | **Third trimester** | **Birth** |  |  |
| **Total bisphenols** |  |  |  |  | | | | |  | | | |  |  |
| First trimester | 0.03 (-0.04, 0.09) | -0.02 (-0.08, 0.05) | 0.06 (-0.05, 0.16) | 0.04 (-0.02, 0.10) | | 0.04 (-0.03, 0.10) | 0.06 (-0.03, 0.15) | | 0.04 (-0.02, 0.10) | | 0.03 (-0.03, 0.09) | 0.02 (-0.05, 0.08) |  |  |
| Second trimester | -0.02 (-0.08, 0.04) | -0.03 (-0.09, 0.03) | -0.01 (-0.11, 0.09) | 0.02 (-0.04, 0.08) | | -0.03 (-0.09, 0.03) | -0.04 (-0.13, 0.05) | | -0.01 (-0.07, 0.04) | | -0.01 (-0.07, 0.05) | -0.03 (-0.09, 0.03) |  |  |
| Third trimester |  | -0.06 (-0.12, 0.01) | -0.04 (-0.15, 0.06) |  | | -0.03 (-0.09, 0.03) | -0.01 (-0.10, 0.08) | |  | | -0.00 (-0.06, 0.06) | -0.04 (-0.10, 0.03) |  |  |
| **Bisphenol A** |  |  |  |  | |  |  | |  | |  |  |  |  |
| First trimester | 0.00 (-0.06, 0.07) | -0.03 (-0.10, 0.03) | 0.07 (-0.04, 0.18) | 0.06 (-0.01, 0.12) | | 0.06 (-0.01, 0.12) | 0.08 (-0.01, 0.17) | | 0.03 (-0.03, 0.09) | | 0.02 (-0.05, 0.08) | 0.00 (-0.06, 0.07) |  |  |
| Second trimester | -0.03 (-0.09, 0.03) | -0.03 (-0.09, 0.03) | -0.01 (-0.11, 0.09) | 0.01 (-0.05, 0.07) | | -0.03 (-0.09, 0.03) | -0.04 (-0.13, 0.05) | | -0.01 (-0.07, 0.04) | | -0.01 (-0.07, 0.05) | -0.03 (-0.09, 0.03) |  |  |
| Third trimester |  | -0.06 (-0.12,  -0.01)* | -0.03 (-0.13, 0.08) |  | | -0.04 (-0.09, 0.02) | 0.03 (-0.06, 0.11) | |  | | -0.01 (-0.07, 0.05) | -0.05 (-0.11, 0.01) |  |  |

Values are regression coefficients (95% confidence intervals) that reflect the differences in fetal head circumference, length and weight in SDS in the second and third trimester and at birth for an interquartile range increase in each natural log-transformed trimester-specific bisphenols (in µmol/g creatinine). Mutually adjusted models include bisphenol concentrations in first, second and third trimester, fetal sex (except in the models for the outcomes at birth), maternal age, pre-pregnancy body mass index, educational level, ethnicity, parity, smoking habits and alcohol consumption (overall during pregnancy), daily caloric intake and folic acid supplement use. Mutually adjusted models were not performed for bisphenols S and F since >80% of concentrations are below the limit of detection in at least one trimester. *p-value<0.05. SDS, standard deviation scores.

**Supplemental Table S5.** Maternal trimester-specific bisphenol concentrations (categorized as detected and undetected) and fetal growth measures

|  | **Fetal head circumference**  **Difference in SDS (95% Confidence Interval)** | | |  | **Fetal length**  **Difference in SDS (95% Confidence Interval)** | | |  | | **Fetal weight**  **Difference in SDS (95% Confidence Interval)** | | | |  |
| --- | --- | --- | --- | --- | --- | --- | --- | --- | --- | --- | --- | --- | --- | --- |
|  | **Second trimester** | **Third trimester** | **Birth** | **Second trimester** | | **Third trimester** | **Birth** | | **Second trimester** | | **Third trimester** | **Birth** |  |  |
| **Bisphenol A** |  |  |  |  | | | | |  | | | |  |  |
| First trimester | -0.05 (-0.17, 0.07) | -0.09 (-0.21, 0.03) | -0.00 (-0.21, 0.20) | 0.12 (0.01, 0.24)* | | 0.03 (-0.09, 0.15) | -0.00 (-0.18, 0.18) | | 0.05 (-0.07, 0.16) | | -0.04 (-0.16, 0.08) | -0.00 (-0.13, 0.12) |  |  |
| Second trimester | -0.01 (-0.20, 0.19) | -0.03 (-0.22, 0.16) | -0.03 (-0.35, 0.29) | 0.04 (-0.15, 0.23) | | -0.00 (-0.20, 0.20) | -0.10 (-0.38, 0.18) | | -0.04 (-0.22, 0.14) | | -0.03 (-0.21, 0.17) | 0.06 (-0.15, 0.26) |  |  |
| Third trimester |  | -0.09 (-0.25, 0.07) | -0.06 (-0.33, 0.22) |  | | 0.00 (-0.16, 0.16) | 0.18 (-0.07, 0.42) | |  | | 0.03 (-0.14, 0.18) | -0.01 (-0.18, 0.16) |  |  |
| **Bisphenol S** |  |  |  |  | |  |  | |  | |  |  |  |  |
| First trimester | 0.17 (0.06, 0.27)† | 0.14 (0.04, 0.25)† | 0.13 (-0.05, 0.30) | 0.10 (0.00, 0.20) | | 0.10 (-0.01, 0.20) | 0.11 (-0.05, 0.26) | | 0.13 (0.03, 0.23)† | | 0.17 (0.07, 0.28)† | 0.12 (0.01, 0.23)† |  |  |
| Second trimester | 0.09 (-0.02, 0.20) | 0.07 (-0.04, 0.17) | 0.07 (-0.11, 0.25) | 0.02 (-0.08, 0.13) | | 0.03 (-0.08, 0.14) | 0.12 (-0.03, 0.28) | | -0.04 (-0.14, 0.06) | | 0.02 (-0.09, 0.12) | 0.11 (-0.00, 0.22) |  |  |
| Third trimester |  | NA | NA |  | | NA | NA | |  | | NA | NA |  |  |
| **Bisphenol F** |  |  |  |  | |  |  | |  | |  |  |  |  |
| First trimester | 0.03 (-0.07, 0.13) | 0.08 (-0.03, 0.17) | -0.04 (-0.21, 0.13) | 0.03 (-0.06, 0.13) | | 0.09 (-0.01, 0.19) | 0.06 (-0.09, 0.21) | | 0.07 (-0.02, 0.17) | | 0.12 (0.02, 0.21)* | 0.12 (0.02, 0.23)† |  |  |
| Second trimester | NA | NA | NA | NA | | NA | NA | | NA | | NA | NA |  |  |
| Third trimester |  | 0.04 (-0.07, 0.15) | -0.05 (-0.23, 0.13) |  | | 0.03 (-0.07, 0.14) | 0.17 (0.01, 0.33)* | |  | | 0.07 (-0.04, 0.18) | 0.09 (-0.02, 0.20) |  |  |

Values are regression coefficients (95% confidence intervals) that reflect the differences in fetal head circumference, length and weight in SDS in the second and third trimester and at birth for detected bisphenol concentrations, as compared to the reference group (undetected concentrations). Models include fetal sex (except in the models for the outcomes at birth). *p-value<0.05, †p-value<0.020

NA, not applicable due to >80% of concentrations below the limit of detection; SDS, standard deviation scores.**Supplemental Table S6.** Maternal pregnancy-averaged and trimester-specific bisphenol concentrations and preterm birth and small size for gestational age

|  | **Preterm birth**  **Odds Ratio (95% Confidence Interval)** | | | | **Small size for gestational age at birth**  **Odds Ratio (95% Confidence Interval)** | | | | |  |
| --- | --- | --- | --- | --- | --- | --- | --- | --- | --- | --- |
|  | **Pregnancy-averaged** | **First trimester** | **Second Trimester** | **Third trimester** | | **Pregnancy-averaged** | **First trimester** | **Second Trimester** | **Third trimester** | |
| **Total bisphenols** | 1.00 (0.66, 1.50) | 1.08 (0.70, 1.65) | 0.93 (0.61, 1.41) | 0.83 (0.54, 1.28) | | 1.06 (0.84, 1.34) | 0.80 (0.62, 1.04) | 1.10 (0.87, 1.40) | 1.24 (0.97, 1.58) | |
| **Bisphenol A** | 1.01 (0.68, 1.50) | 1.06 (0.68, 1.64) | 0.93 (0.62, 1.40) | 0.89 (0.59, 1.34) | | 1.10 (0.88, 1.37) | 0.92 (0.71, 1.20) | 1.11 (0.88, 1.40) | 1.19 (0.94, 1.52) | |
| **Bisphenol S** | 1.04 (0.67, 1.63) | 0.96 (0.59, 1.56) | 0.90 (0.58, 1.38) | NA | | 0.76 (0.58, 1.00) | 0.72 (0.54, 0.96)* | 0.97 (0.76, 1.25) | NA | |
| **Bisphenol F** | 0.87 (0.54, 1.41) | 1.00 (0.62, 1.59) | NA | 0.90 (0.56, 1.44) | | 0.95 (0.72, 1.25) | 0.83 (0.62, 1.10) | NA | 1.09 (0.84, 1.42) | |

Values are odds ratios (95% confidence intervals) from binary logistic regression models that reflect the risk of preterm birth and small size for gestational age at birth for an interquartile range increase in each natural log-transformed pregnancy-averaged and trimester-specific bisphenols (in µmol/g creatinine). Models include maternal age, pre-pregnancy body mass index, ethnicity, educational level, parity, folic acid supplement use, alcohol consumption and smoking habits during each trimester or during pregnancy and daily caloric intake. *p-value<0.05

NA, not applicable due to >80% of concentrations below the limit of detection.

**Supplemental Table S7.** Maternal trimester-specific bisphenol concentrations and preterm birth and small size for gestational age at birth, mutually adjusted model

|  | **Preterm birth**  **Odds Ratio (95% Confidence Interval)** | | |  | | **Small size for gestational age at birth**  **Odds Ratio (95% Confidence Interval)** | | |
| --- | --- | --- | --- | --- | --- | --- | --- | --- |
|  | **First trimester** | **Second Trimester** | **Third trimester** | | **First trimester** | | **Second Trimester** | **Third trimester** |
| **Total bisphenols** | 1.08 (0.70, 1.66) | 0.93 (0.61, 1.42) | 0.83 (0.54, 1.28) | | 0.80 (0.02, 1.04) | | 1.11 (0.88, 1.40) | 1.24 (0.97, 1.58) |
| **Bisphenol A** | 1.06 (0.68, 1.65) | 0.93 (0.62, 1.41) | 0.89 (0.59, 1.35) | | 0.92 (0.71, 1.19) | | 1.11 (0.88, 1.40) | 1.20 (0.94, 1.51) |

Values are odds ratios (95% confidence intervals) from binary logistic regression models that reflect the risk of preterm birth and small size for gestational age at birth for an interquartile range increase in each natural log-transformed bisphenol (in µmol/g creatinine). Mutually adjusted models include bisphenol concentrations in first, second and third trimester, maternal age, pre-pregnancy body mass index, ethnicity, educational level, parity, folic acid supplement use, alcohol consumption and smoking habits (overall during pregnancy) and daily caloric intake. Mutually adjusted models were not performed for bisphenols S and F since >80% of concentrations are below the limit of detection in at least one trimester.

**Supplemental Table S8.** Maternal trimester-specific bisphenol concentrations (categorized as detected and undetected) and preterm birth and small size for gestational age at birth

|  | **Preterm birth**  **Odds Ratio (95% Confidence Interval)** | | | **Small size for gestational age at birth**  **Odds Ratio (95% Confidence Interval)** | | |
| --- | --- | --- | --- | --- | --- | --- |
|  | **First trimester** | **Second Trimester** | **Third trimester** | **First trimester** | **Second Trimester** | **Third trimester** |
| **Bisphenol A** | 1.52 (0.72, 3.20) | 1.78 (0.61, 5.14) | 1.16 (0.40, 3.32) | 0.95 (0.601, 1.52) | 1.01 (0.48, 2.15) | 1.11 (0.58, 2.13) |
| **Bisphenol S** | 1.61 (0.81, 3.17) | 0.91 (0.44, 1.87) | NA | 0.55 (0.38, 0.82)† | 0.88 (0.57, 1.36) | NA |
| **Bisphenol F** | 1.14 (0.57, 2.28) | NA | 1.03 (0.49, 2.16) | 0.54 (0.35, 0.83)† | NA | 0.86 (0.56, 1.33) |

Values are odds ratios (95% confidence intervals) from binary logistic regression models that reflect the risk of preterm birth and small size for gestational age at birth for detected bisphenol concentrations, as compared to the reference group (undetected concentrations). Unadjusted models are presented. †p-value<0.020

NA, not applicable due to >80% of concentrations below the limit of detection.

**Supplemental Table S9.** Maternal pregnancy-averaged and trimester-specific bisphenol concentrations and gestational age at birth

|  | **Gestational age at birth**  **Difference in weeks (95% Confidence Interval)** | | | | | | |
| --- | --- | --- | --- | --- | --- | --- | --- |
|  | **Continuous bisphenol concentrations** | | | | **Detected and undetected bisphenol concentrations** | | |
|  | **Pregnancy-averaged** | **First trimester** | **Second trimester** | **Third trimester** | **First trimester** | **Second trimester** | **Third trimester** |
| **Total bisphenols** | -0.05 (-0.14, 0.05) | -0.06 (-0.16, 0.04) | -0.03 (-0.13, 0.06) | 0.02 (-0.07, 0.12) | - | - | - |
| **Bisphenol A** | -0.05 (-0.14, 0.04) | -0.02 (-0.12, 0.08) | -0.03 (-0.12, 0.06) | -0.01 (-0.10, 0.08) | 0.22 (0.03, 0.41)* | -0.06 (-0.36, 0.24) | 0.00 (-0.25, 0.25) |
| **Bisphenol S** | NA | -0.05 (-0.16, 0.06) | 0.05 (-0.04, 0.14) | NA | 0.05 (-0.11, 0.21) | 0.02 (-0.15, 0.19) | NA |
| **Bisphenol F** | NA | -0.08 (-0.19, 0.02) | NA | 0.07 (-0.04, 0.17) | 0.03 (-0.13, 0.18) | NA | 0.07 (-0.10, 0.24) |

Values are regression coefficients (95% confidence intervals) that reflect the differences in gestational age at birth in weeks for an interquartile range increase in each natural log-transformed pregnancy-averaged and trimester-specific bisphenol concentration and for detected bisphenol concentrations, as compared to the reference group (undetected concentrations). Models include maternal age, pre-pregnancy body mass index, ethnicity, educational level, parity, folic acid supplement use, alcohol consumption and smoking habits during each trimester and overall during pregnancy and daily caloric intake. *p-value<0.05

NA, not applicable due to >80% of concentrations below the limit of detection.

**Supplemental Figure S1.** Selection of study participants

Mothers of singleton live-born children

with information on exposure to

bisphenols during pregnancy

**N = 1,405**

N = 26 excluded, due to missing information on

bisphenols in at least one trimester of pregnancy

First trimester: n = 17

Second trimester: n = 15

Third trimester: n = 14

Mothers of singleton live-born children

with information on exposure to

bisphenols in all trimesters of pregnancy

**N= 1,379**

**Total population for analysis** N=1,379

**Second trimester**
Head circumference: n = 1,362
Femur length: n = 1,369
Estimated fetal weight: n = 1,366

**Third trimester**
Head circumference: n = 1,369
Femur length: n = 1,377
Estimated fetal weight: n = 1,375

**Birth**
Gestational age: n = 1,379
Weight: n = 1,379
Head circumference: n = 835
Length: n = 975

**Supplemental Figure S2.** Directed acyclic graph depicting the relationship between exposure to bisphenols, fetal growth and birth outcomes and covariates

Exposure: Bisphenols

Outcome: Fetal Growth and birth outcomes

Potential effect modifiers:

Folic acid supplement use

Fetal sex

Potential confounders:

Maternal ethnicity

Maternal age

Maternal pre-pregnancy body mass index

Parity

Maternal education

Alcohol and smoking habits during pregnancy

Maternal daily caloric intake

Folic acid supplement use

Fetal sex
